# Ventricular Fibrillation Is a Sign of Life

**DOI:** 10.21470/1678-9741-2021-0428

**Published:** 2023-09-19

**Authors:** Nabil Dib, Raphael Martins, Erwan Flécher

**Affiliations:** 1 Division of Thoracic, Cardiac and Vascular Surgery, Pontchaillou University Hospital, Rennes, France; 2 Division of Cardiology, Pontchaillou University Hospital, Rennes, France

**Keywords:** Biological Phenomena, Cardiovascular Diseases, Physiological Phenomena, Ventricular Fibrillation

## Abstract

Ventricular fibrillation (VF) is a deadly rhythm problem. With asystole, it represents one of the most extreme emergencies that may engage vital prognosis within only few minutes if appropriated treatment is not instituted. It is learned in all medical schools worldwide that VF is not compatible with consciousness and sustained life. Moreover, at 37°C, and without restauration of cardiac flow, VF may be responsible for severe and most often irreversible brain damage after 3 minutes.

Herein we report an exceptional case of a patient who presented with authentic ventricular fibrillation (VF) for many hours and who remained perfectly conscious and walking into our hospital^[1,2,3]^.

A 57-year-old male with previous Hodgkin's lymphoma had complications related to radiotherapy. Radiotherapy-induced coronary lesions were clinically treated, and severe aortic stenosis justified transcatheter aortic valve implantation. The latter required a pacemaker implantation due to a high-degree atrioventricular block. After temporary clinical improvement, refractory heart failure occurred. Heart transplantation was not indicated and a left ventricular assist device (Jarvik® Heart Inc., NY) (Figure 1) was implanted in destination therapy. Three years later, the patient was hospitalized for asthenia and anemia. On admission, VF was discovered although the patient was alert and ambulatory (Video).

**Fig. 1:**
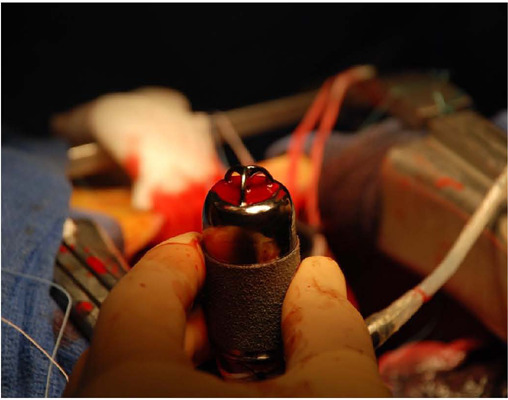
Jarvik® 2000 before implantation.

**Video:**
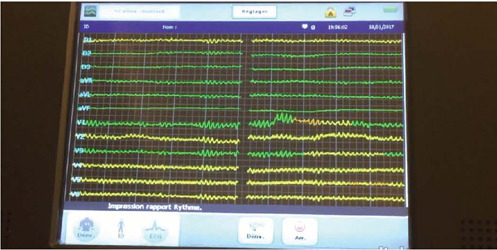
Evidence of ventricular fibrillation in a male speaking patient. Link: https://s3.sa-east-1.amazonaws.com/publisher.gn1.com.br/bjcvs.org/videos/e20210428.mp4

The pacemaker examination revealed 36 hours of continued VF. An electric shock restored sinus rhythm and a defibrillator was implanted in place of the pacemaker. Clinical condition of the patient dramatically improved, and he was promptly discharged. He died from septic shock 3 months later.

## Abbreviations, Acronyms & Symbols

VF: Ventricular fibrillation
